# Comparative Analysis of Clinical Outcomes of High-power, Short-duration Ablation versus Low-power, Long-duration Ablation Strategy in Patients with Atrial Fibrillation: A Comprehensive Umbrella Review of Meta-analyses

**DOI:** 10.19102/icrm.2024.15083

**Published:** 2024-08-15

**Authors:** Peddi Pavani, Olusegun Abiola Olanrewaju, Raja Subhash Sagar, Monika Bai, Jai Chand, Vishal Bhatia, Fnu Sagar, Fnu Karishma, Hamza Islam, Aman Kumar, Fnu Versha, Rabia Islam, Taha Nadeem

**Affiliations:** 1 Department of Surgery, Kurnool Medical College, Kurnool, India; 2 Department of Medicine, Stavropol State Medical University, Stavropol, Russia; 3 Department of Medicine, Liaquat University of Medical and Health Sciences, Jamshoro, Pakistan; 4 Department of Medicine, Peoples University of Medical and Health Sciences for Women, Nawabshah, Pakistan; 5 Department of Medicine, Khairpur Medical College, Khairpur, Pakistan; 6 Department of Medicine, Bahria University Medical and Dental College, Karachi, Pakistan; 7 Department of Medicine, Ghulam Muhammad Mahar Medical College, Sukkur, Pakistan; 8 Department of Medicine, Punjab Medical College, Faisalabad, Pakistan; 9 Department of Medicine, Allama Iqbal Medical College, Lahore, Pakistan

**Keywords:** Atrial fibrillation, high-power short duration, low-power long duration, radiofrequency ablation, short duration

## Abstract

Atrial fibrillation (AF) affects around 33 million people worldwide, rendering it a common cardiac arrhythmia. Catheter ablation (CA) has evolved as a leading therapeutic intervention for symptomatic AF. This umbrella review systematically evaluates existing systematic reviews and meta-analyses to assess the safety, efficacy, and potential of high-power, short-duration (HPSD) ablation as an alternative therapy option for AF. A thorough exploration was undertaken across PubMed, the Cochrane Library, and Embase to identify pertinent studies for inclusion in this umbrella review. The Grading of Recommendations Assessment, Development, and Evaluation (GRADE) method was employed to assess the overall certainty of the evidence comprehensively, and the quality of the incorporated reviews was meticulously evaluated through use of the AMSTAR 2 tool, the Cochrane Collaboration tool, and the Newcastle–Ottawa scale. In this study, we initially identified 35 systematic reviews and meta-analyses, narrowing them down to a final selection of 11 studies, which collectively integrated data from 6 randomized controlled trials and 26 observational studies. For primary efficacy outcomes, the HPSD approach led to a non-significant decrease in the risk of atrial tachyarrhythmia recurrence (risk ratio [RR], 0.88; 95% confidence interval [CI], 0.70–1.12; *I*^2^ = 90%; *P* = .31) and a significantly reduced risk of AF recurrence (RR, 0.53; 95% CI, 0.42–0.67; *I*^2^ = 0%; *P* < .00001) compared to the low-power, long-duration (LPLD) approach. In terms of primary safety outcomes, the HPSD approach significantly reduced the risk of esophageal thermal injury (ETI) (RR, 0.71; 95% CI, 0.61–0.83; *I*^2^ = 0%; *P* < .00001) and facilitated a non-significant decrease in the risk of other major complications (RR, 0.87; 95% CI, 0.73–1.03; *I*^2^ = 0%; *P* = .10). In conclusion, HPSD therapy is safer and more effective than LPLD therapy, facilitating decreased AF recurrence rates along with reductions in ETI, total procedure duration, ablation number, ablation time, fluoroscopy time, and acute pulmonary vein reconnection.

## Introduction

Globally, approximately 33 million individuals suffer from atrial fibrillation (AF), the most prevalent abnormal heartbeat observed in clinical practice.^1^ It is frequently associated with complications such as strokes, heart failure, and an increased risk of mortality. This disorder is a significant concern in medicine because of its widespread prevalence and link to serious health problems.^1^ Catheter ablation (CA) for AF has progressed from a trial approach to the most effective therapy for those with symptoms. This change is supported by positive clinical outcomes data, demonstrating that this treatment has potential effects for patients suffering from AF-related issues.^2^

Historically, doctors employed radiofrequency (RF) energy to induce low-power, long-duration (LPLD) lesions in the heart during CA, guided by parameters such as the force–time integral.^3^ However, the optimum ablation settings for safe long-term pulmonary vein isolation (PVI) are still being investigated. There is an increasing interest in creating high-power, short-duration (HPSD) lesions, which appear to shorten the procedure and fluoroscopy time and potentially lower the risk of adverse effects by limiting heat transmission to nearby tissues and causing less damage.^4^

Many ablation centers have traditionally used the LPLD approach to generate lesions for PVI.^5^ This strategy seeks to reduce the likelihood of problems, such as cardiac tamponade and pulmonary vein stenosis. Typically, these settings require applying 25–40 W of power for 20–40 s at each location, with a contact force (CF) of 10–20 g.^6^ However, there needs to be a clear understanding of what defines HPSD ablation, with research indicating that power levels range from 50–90 W and durations vary across investigations.^7^ Considering the gap in the literature and universal implementation of HPSD instead of LPLD, we performed an umbrella review of all available systematic reviews and meta-analyses to determine the safety, effectiveness, and potential of this alternate therapy option for HPSD ablation.

## Methodology

This comprehensive review adhered to the guidelines outlined in the Preferred Reporting Items for Systematic Reviews and Meta-analyses and the Cochrane Collaboration Handbook.^8,9^

### Search strategy

The search strategy was executed across various databases, including PubMed, the Cochrane Library, and Embase, ensuring a thorough exploration of the scientific literature. An extensive array of keywords and Medical Subject Headings was carefully curated to guarantee inclusivity. Encompassing facets such as “radiofrequency ablation,” “high-power,” “short-duration,” “low-power,” “long-duration,” “atrial fibrillation treatment,” “efficacy,” “safety profiles,” “catheter ablation,” “heart rhythm disorders,” and “cardiac arrhythmias,” these keywords were strategically combined using the Boolean operators “AND” and “OR” to both refine and broaden the search. A summary of the detailed search strategy, including specific combinations of keywords and operators, can be found in **Supplementary Table S1**. To uphold the integrity of the process and minimize potential selection bias, two independent researchers searched the literature, resolving disagreements through consensus. A third researcher was engaged in cases of persistent discrepancies to ensure resolution and reliability.

### Study inclusion and exclusion criteria

#### Inclusion criteria

This umbrella review centered on systematically executed, high-quality reviews and meta-analyses, aiming to investigate the comparative efficacy of HPSD and LPLD for the management of AF. The encompassed studies targeted adult participants aged ≥18 years conclusively diagnosed with AF. The review incorporated studies exploring HPSD RF ablation (RFA) as a therapeutic approach for AF, including meta-analyses and systematic reviews directly comparing the effectiveness and safety of HPSD versus LPLD in treating AF. Inclusion criteria extended to studies providing valuable insights into pertinent clinical outcomes, such as atrial tachyarrhythmias (ATAs), AF recurrence, esophageal thermal injury (ETI), and other significant complications.

#### Exclusion criteria

This umbrella review excluded primary studies, conference abstracts, letters, editorials, and reviews failing to meet the stringent criteria for systematic reviews and meta-analyses. Additionally, reviews focusing on pediatric populations (participants aged <18 years) or using animal models were expressly omitted. Studies investigating treatments unrelated to HPSD or LPLD for AF were not considered. Furthermore, reviews that did not directly compare HPSD with LPLD within AF were excluded. This comprehensive review rigorously maintained exclusion criteria, ensuring the exclusion of studies lacking relevant clinical outcomes or reporting incomplete data.

### Data extraction and definitions

Relevant information was extracted during the data-extraction process, encompassing publication details, study attributes, participant features, ablation procedure strategies, and clinical outcomes. Primary efficacy outcomes included ATA and AF recurrence post-blanking (2 or 3 months post-ablation), depending on the studies included, whereas primary safety outcomes were ETI and other major complications. Secondary outcomes included first-pass pulmonary vein isolation (FPI), acute pulmonary vein reconnection (PVR), procedural time, PVI number, ablation number, and fluoroscopy time. High power was defined as >40 W, and the extracted data were separated into the high-power (HP) group and the low-power (LP) group.

ATA recurrence was defined by symptomatic or asymptomatic ATAs lasting >30 s after the blanking period post-ablation. ETI was defined as esophageal thermal injury brought on by ablation, evaluated through endoscopy and/or magnetic resonance imaging late gadolinium enhancement. FPI was defined as the first-pass RF-delivery PVI achievement rate, and acute PVR was defined as the pulmonary vein electrical reconnection rate after the first-pass CA. Ablation number refers to the count or quantity of RFAs performed during the procedure. Procedural time was defined as the time between the beginning of anesthesia and removal of all sheaths. In contrast, fluoroscopy time was the total time spent using a fluoroscope during the procedure.

### Assessment of the risk of bias

The methodological quality of the incorporated reviews and meta-analyses underwent a meticulous evaluation by two independent investigators using the AMSTAR 2 tool. This tool comprehensively addresses 16 essential methodological domains, offering a nuanced assessment. The overall quality of the studies emerged was categorized as either high, moderate, low, or critically low, guided by established criteria.^10^

We employed the Cochrane Collaboration risk-of-bias tool to assess the risk of bias inherent in randomized controlled trials (RCTs) included in individual meta-analyses.^11^ This comprehensive tool systematically evaluates eight potential sources of bias, including random sequence generation, allocation concealment, blinding of participants and evaluators, outcome assessments, and handling of incomplete outcome data. The quality of observational studies underwent scrutiny by application of the Newcastle–Ottawa scale. This assessment encompassed crucial domains, such as study design, participant selection, blinding, outcome reporting, and other pertinent parameters, ensuring a comprehensive evaluation of their robustness.^12^

The certainty of evidence and the strength of recommendations derived from meta-analyses underwent rigorous scrutiny using the Grading of Recommendations Assessment, Development, and Evaluation (GRADE) method.^13^ This method categorizes evidence into four tiers: “high,” “moderate,” “low,” and “very low.” Initiated at the “high” level, the GRADE assessment underwent subsequent adjustments based on identified risks of bias, results inconsistency, evidence indirectness, imprecision, or publication bias. Two researchers conducted the GRADE assessment independently for each study’s primary outcomes, engaging in discussions and reaching agreements to address any disparities.

### Statistical analysis

All statistical analyses were meticulously executed, using STATA 16 and Review Manager version 5.4. Categorical outcomes were assessed through the computation of risk ratios (RRs) accompanied by 95% confidence intervals (CIs), employing the DerSimonian and Laird random-effects model. Mean differences were calculated for continuous data, with statistical significance in two-sided tests set at *P* < .05. The *I*^2^ statistic was applied to scrutinize heterogeneity among study associations.^14^ Rigorous sensitivity analyses were performed to assess the robustness of summary estimates and pinpoint any singular study significantly contributing to heterogeneity, notably when heterogeneity exceeded 75%. Egger’s regression asymmetry test was applied to primary outcomes, investigating evidence of small-study effects,^15^ where *P* < .05 indicated such effects. “P-hacking”^16^ and evaluating publication bias were conducted through funnel plots of primary outcomes.

Regarding ethical considerations and conflicts of interest, this umbrella review exclusively relies on previously published systematic reviews and meta-analyses, eliminating the need to collect or analyze primary data from human participants. Consequently, ethical review board approval and patient consent did not apply to this study. The authors unequivocally assert that no conflicts of interest, whether financial or non-financial, could sway the impartiality or interpretation of the findings in this umbrella review. The entire research process and outcomes remain impervious to external affiliations or funding sources, ensuring a commitment to unbiased reporting.

## Results

### Study selection

Initially, a total of 35 systematic reviews and meta-analyses were identified, and subsequent elimination of duplicate entries was undertaken. Upon exhaustive scrutiny of the complete texts, a final selection of 11 systematic reviews and meta-analyses^17–27^ was made. These chosen studies comprehensively compiled data from 6 RCTs and 26 observational studies, encompassing prospective and retrospective cohort studies. **Table 1** briefly outlines the key characteristics of the integrated meta-analyses within the scope of this review.

### Risk of bias of included studies

**Supplementary Figure S1:**
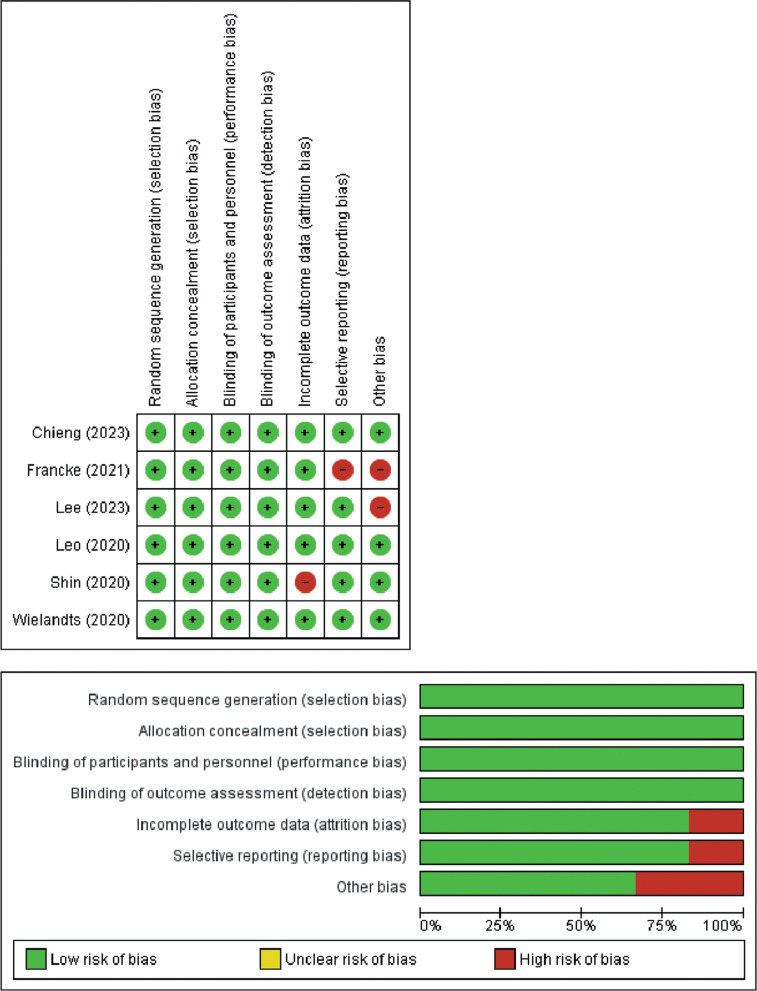
Cochrane risk-of-bias assessment for individual randomized controlled trials.

The methodological quality assessments for the 11 systematic reviews and meta-analyses, appraised through the AMSTAR 2 tool, are delineated in **Supplementary Table S2**. Each of the 11 studies was assigned a moderate-quality rating. The GRADE assessment, detailed in **Supplementary Table S3**, indicated a diverse range of certainty levels within the reviews employed for our study, encompassing evaluations from low to high. Individual RCTs underwent a comprehensive quality evaluation using the Cochrane risk-of-bias tool, revealing trials characterized by a moderate to low risk of bias, as illustrated in **Supplementary Figure S1**. Furthermore, the quality appraisal of observational studies was meticulously executed using the Newcastle–Ottawa scale, exposing a spectrum of quality across the studies included in the analysis, extending from fair to good, as expounded in **Supplementary Table S4**.

### Synthesis of results

#### Primary efficacy outcomes

**Figure 1:**
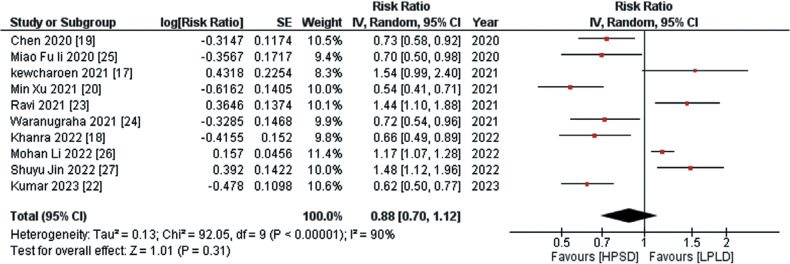
Recurrence of atrial tachyarrhythmias. This figure presents a comprehensive analysis of the recurrence of atrial tachyarrhythmias after radiofrequency ablation, revealing a non-significant decrease in the risk of atrial tachyarrhythmia recurrence associated with the high-power, short-duration approach compared to the low-power, long-duration approach. *Abbreviations:* CI, confidence interval; HPSD, high-power, short-duration; IV, inverse variance; LPLD, low-power, long-duration; RR, risk ratio; SE, standard error.

**Figure 2:**
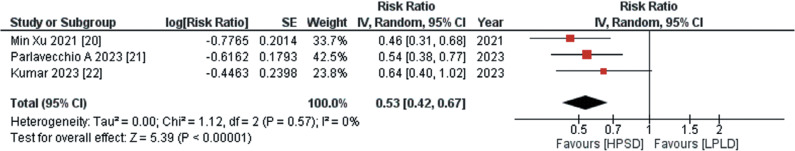
Recurrence of atrial fibrillation following ablation. This figure illustrates a pooled analysis that reveals a statistically significant reduction in the risk of atrial fibrillation recurrence associated with the high-power, short-duration approach compared to the low-power, long-duration approach. *Abbreviations:* CI, confidence interval; HPSD, high-power, short-duration; IV, inverse variance; LPLD, low-power, long-duration; RR, risk ratio; SE, standard error.

The primary efficacy outcomes encompassed the recurrence of ATAs and AF. Across 10 out of 11 studies, data on ATA recurrence were reported. The pooled analysis indicated a non-significant decrease in the risk of ATA recurrence associated with the HPSD approach compared to the LPLD approach (RR, 0.88; 95% CI, 0.70–1.12; *I*^2^= 90%; *P* = .31), as illustrated in **Figure 1**. In response to the notable in-study heterogeneity, a leave-one-out sensitivity analysis was conducted, revealing that no individual study was the cause of the observed high heterogeneity. The data about AF recurrence, sourced from 3 out of the 11 studies, indicated through pooled analysis that the employment of the HPSD approach was associated with a significantly reduced risk of AF recurrence compared to the LPLD approach (RR, 0.53; 95% CI, 0.42–0.67; *I*^2^ = 0%; *P* < .00001), as depicted in **Figure 2**.

#### Primary safety outcomes

**Figure 3:**
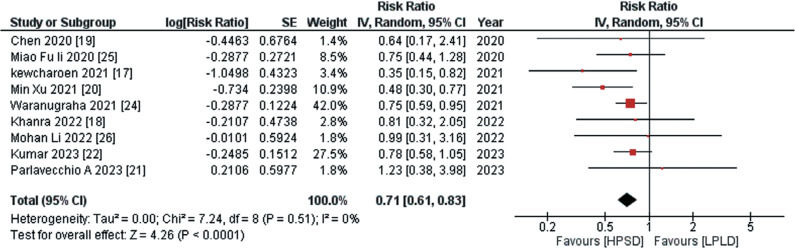
Risk of esophageal thermal injury following ablation. This figure presents an aggregated analysis that reveals a statistically significant reduction in the risk of esophageal thermal injury associated with the high-power, short-duration approach compared to the low-power, long-duration approach. *Abbreviations:* CI, confidence interval; HPSD, high-power, short-duration; IV, inverse variance; LPLD, low-power, long-duration; RR, risk ratio; SE, standard error.

**Figure 4:**
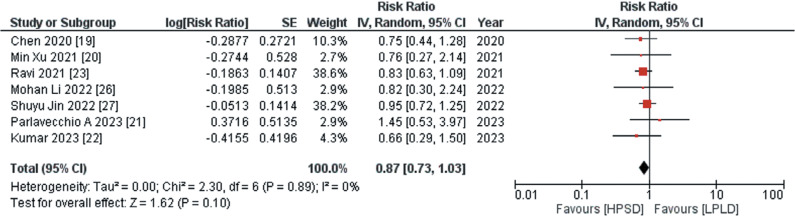
Risk of other major complications following ablation. This figure elucidates the pooled analysis investigating the incidence of other major complications following radiofrequency ablation. The pooled analysis portrays that treatment with the high-power, short-duration approach was associated with a non-significant decrease in the risk of other major complications compared to treatment with the low-power, long-duration approach. *Abbreviations:* CI, confidence interval; HPSD, high-power, short-duration; IV, inverse variance; LPLD, low-power, long-duration; RR, risk ratio; SE, standard error.

The primary safety endpoints encompassed ETI and other major complications. Data regarding ETI were provided by 9 out of the 11 studies, and the aggregated analysis demonstrated that use of the HPSD approach was associated with a significantly reduced risk of ETI compared to the LPLD approach (RR, 0.71; 95% CI, 0.61–0.83; *I*^2^ = 0%; *P* < .00001), as depicted in **Figure 3**. The data pertaining to other major complications, sourced from 7 out of the 11 studies, showed through pooled analysis that the adoption of the HPSD approach was associated with a non-significant decrease in the risk of other major complications compared to the LPLD approach (RR, 0.87; 95% CI, 0.73–1.03; *I*^2^ = 0%; *P* = .10), as illustrated in **Figure 4**.

#### Secondary outcomes

The secondary outcomes included FPI, total procedure time, ablation time, ablation number, fluoroscopy time, and acute PVR. The results of the analysis of secondary outcomes are summarized in **Table 2**. The pooled analysis revealed that treatment with the HPSD approach was associated with a significantly increased risk of FPI and a significantly decreased risk of acute PVR. Furthermore, the analysis revealed that treatment with the HPSD approach was associated with a significantly reduced total procedure time, fluoroscopy time, and ablation time compared to the LPLD approach, along with a non-significantly reduced ablation number.

#### P-hacking, publication bias, and small-study effect

**Supplementary Figure S2:**
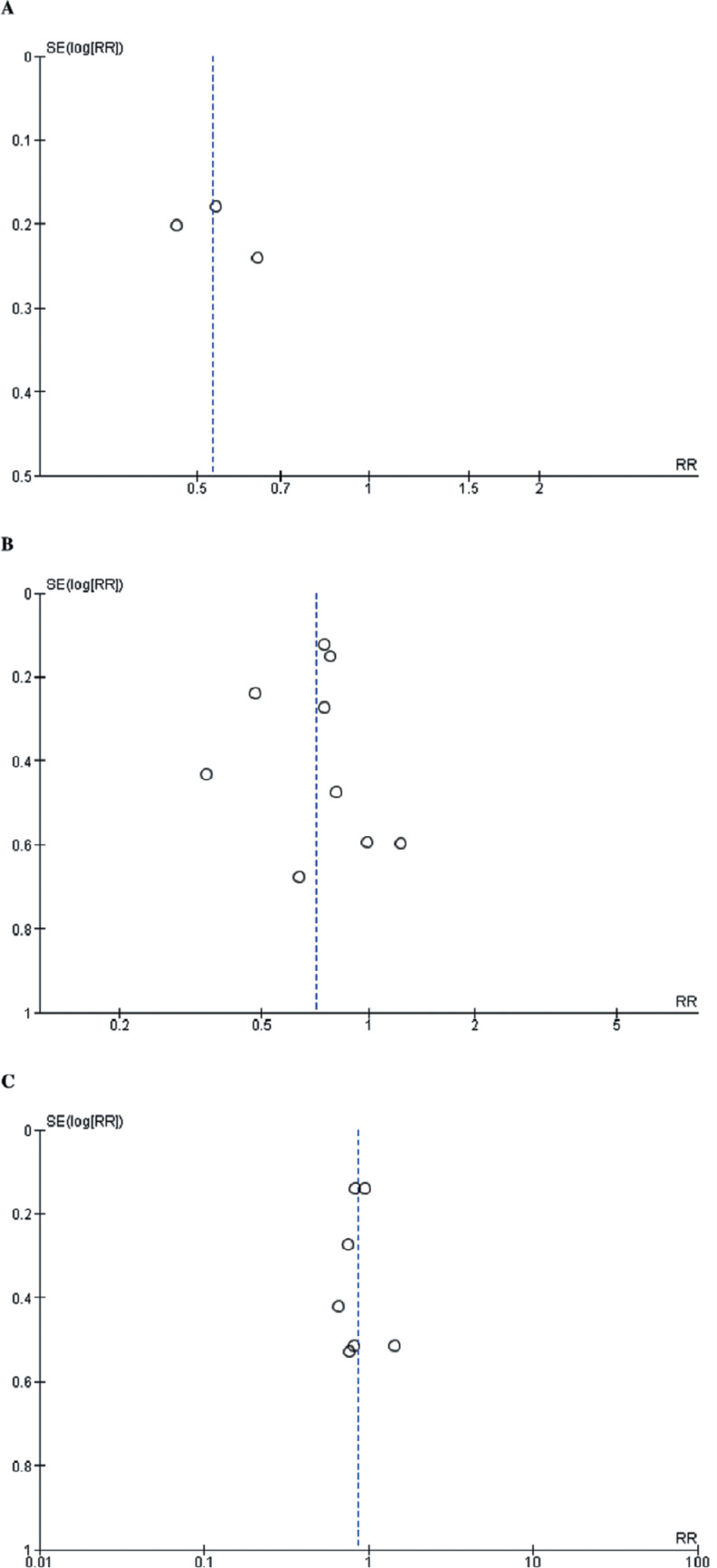
Funnel plots. **A:** Recurrence of atrial tachyarrhythmias. **B:** Esophageal thermal injury. **C:** Other major complications. The funnel plots showed no risk of publication bias.

The absence of evidence indicating *P*-hacking in our study signifies that the results were not manipulated to achieve a predetermined outcome. In scrutinizing the primary outcomes, we conducted a thorough analysis with sufficient studies, facilitating a comprehensive funnel plot analysis. Our findings disclosed a symmetrical distribution of data points in the funnel plots for each primary outcome. The observed symmetry in the data implies the non-existence of publication bias, as demonstrated in **Supplementary Figure S2**. Furthermore, we employed Egger’s regression asymmetry test to assess small-study effects for the primary outcomes, with values for each outcome exceeding 0.05. This indicates a lack of substantial evidence supporting small-study effects. The results of Egger’s regression asymmetry test are briefly outlined in **Table 3**.

## Discussion

In recent decades, percutaneous CA has developed as an essential treatment option for AF, with the goal of restoring and maintaining normal sinus rhythm. PVI is now recognized as the cornerstone of catheter-based ablation therapies for the treatment of paroxysmal and early persistent AF.^28,29^ Recent studies have looked into using greater RF energy output with shorter pulses in AF ablation techniques to improve safety and create longer-lasting lesions.^30^ This HPSD ablation technique uses higher RF power levels (45–50 W) for shorter durations (5–15 s) per RF energy application instead of the traditional method of lower power (25–30 W) for longer application durations (30–60 s).^31^ The HPSD approach uses a short burst of RF energy to heat up and destroy specific tissue in the atrium and avoids heating nearby tissues, such as the esophagus, which lowers the risk of damage to other surrounding tissues.^32^

Our comprehensive umbrella review of 11 systematic reviews and meta-analyses comparing the HPSD method to LPLD sought to identify clinical outcomes that persisted across both procedures. Our primary focus was on the recurrence of ATAs, AF, ETI, and other significant complications. Regarding efficacy outcomes, the study revealed that HPSD was related to a decrease in the probability of ATA recurrence, although this finding was non-significant; nevertheless, the reduced risk of AF recurrence was substantial enough to evaluate this therapy over LPLD clinically. Our findings markedly differ from those of a recent meta-analysis conducted by Li et al.^26^ Their analysis encompassed ATA and AF recurrence rates at 6-month and 12-month intervals post-surgery, collated from 18 studies. Initially, there was no statistically significant difference in AF recurrence rates between the HPSD and LPLD groups (RR, 1.11; 95% CI, 0.96–1.28; *I*^2^ = 45%; *P* = .16). However, with an extended follow-up period of 12 months, a notable divergence emerged. HPSD groups exhibited greater rates of both AF and ATA recurrence compared to conventional groups (for freedom from AF: RR, 1.17; 95% CI, 1.07–1.27; *I*^2^ = 32%; *P* = .0003) (for freedom from AT: RR, 1.11; 95% CI, 1.05–1.17; *I*^2^ = 32%; *P* < .0001). This suggests that the therapeutic benefits of HPSD may become more pronounced with longer-term patient follow-up. Nevertheless, our comprehensive analyses, incorporating all available literature to date, consistently indicate that HPSD exhibits greater clinical efficacy over LPLD, specifically in terms of AF recurrence rather than ATA recurrence.

In the context of safety outcomes, our investigation demonstrated that HPSD therapy exhibited a significantly reduced risk of ETI. However, the reduction in major complications, while notable, did not reach statistical significance, thus requiring further investigation to establish its enhanced safety profile and decreased adverse event risks. A recent study conducted in 2022 by Khanra et al.^18^ yielded similar observations in their pooled analysis. They reported a lower incidence and severity of ETI in the HPSD group. However, the differences did not reach statistical significance. Notably, despite comparable maximum esophageal temperatures between the two groups, the HPSD group experienced significantly fewer esophageal temperature alerts, indicating a potential advantage in terms of safety.

Our study systematically investigated several other important outcomes, consisting of FPI, total procedure time, ablation time, ablation number, fluoroscopy time, and acute PVR. The analysis revealed a noteworthy increase in the incidence of FPI, coupled with significant reductions in total procedure time, ablation number, ablation time, fluoroscopy time, and acute PVR. These findings collectively underscore the statistical significance supporting the superior efficacy of HPSD therapy compared to LPLD in the management of patients with AF. There are two phases involved in the thermal damage caused by an irrigated RFA catheter tip: resistive and conductive. When using HP settings, most RF energy is absorbed in the first 1–3 mm of tissue around the electrode tip of the ablation catheter.^33–35^ By increasing the resistive heating’s rim size to >50°C, HPSD ablation encourages the development of long-lasting lesions. Because the antral thickness of these lesions is often <4 mm, their bigger dimensions and shallower depth make them especially suitable for PVI.^34^ This feature of HPSD ablation helps to reduce the risk of extracardiac damage, improve PVI, and promote contiguous ablation lesions.^35^ Interestingly, HPSD protocols depend less on the conductive phase of heating, which is consistent with our findings of decreased acute PVR and elevated FPI.

Our study has several strengths and some limitations. In terms of strengths, this is the first umbrella review ever conducted on this topic, reducing uncertainties and gaps between previous meta-analyses’ findings. Second, we performed an Egger’s regression test to demonstrate that no publication bias was reported. In terms of limitations, different systematic reviews and meta-analyses have used a variety of evidence-synthesis procedures, such as quality-evaluation tools and meta-analytic methodologies, which may lead to uncertainty. Second, high heterogeneity in some outcomes may be attributed to the diverse methodology, inclusion and exclusion criteria, patient population, study design, and outcome measures employed in all systematic reviews and meta-analyses.

## Conclusion

In conclusion, HPSD therapy emerges as a safer and more successful alternative to traditional LPLD therapy. This method not only reduces AF recurrence rates significantly but also mitigates undesirable consequences, such as ETI. Furthermore, there are considerable improvements in many procedural elements, such as total procedure duration, ablation number, ablation time, fluoroscopy time, and acute PVR. To support these findings, future research should incorporate more extensive clinical studies with more significant sample numbers. This method will give a more solid foundation for evidence-based findings while also improving our understanding of the comparative efficacy and safety of HPSD therapy in AF management.

## Figures and Tables

**Table 1:** Study Characteristics

Author (Year)	No. of Studies Included in Each Meta-analysis	Time Period	Total No. of Patients	No. of Patients		Male (%)	Mapping Tools	Primary Outcomes	Secondary Outcomes
				HPSD Group	LPLD Group				
Kewcharoen et al. (2021)^17^	10	2006–2020	2274	1393	881	74.6	EnSite™, CARTO^®^ 3	Recurrence of atrial arrhythmias, periprocedural complications	Procedure time
Khanra et al. (2022)^18^	20	N/A	3889	2136	1753	N/A	EnSite™, CARTO^®^ 3	Atrial arrhythmia recurrence, first-pass pulmonary vein	Procedural and fluoroscopy time, acute PVRs, ETIs
Chen et al. (2020)^19^	10	N/A	2467	1466	1001	65.3	EnSite™, CARTO^®^ 3	FPI of PVs, recurrence of atrial arrhythmias, major complications	Procedural time, fluoroscopy time, ablation time, ETI
Xu et al. (2022)^20^	15	2016–2019	3255	1856	1399	64.5	EnSite™, CARTO^®^ 3	ATA and AF recurrence post-blanking period and major complications; the latter included ETI and CA-related heart complications such as cardiac tamponade	FPI and acute PVR, procedural time, ablation number for PVI, and fluoroscopy time
Parlavecchio et al. (2023)^21^	5	2020–2023	424	209	215	N/A	EnSite™, CARTO^®^ 3	ATA and AF recurrence post-blanking period and major complications; the latter included ETI and CA-related heart complications such as cardiac tamponade	FPI and acute PVR, procedural time, ablation number for PVI, and fluoroscopy time
Kumar et al. (2023)^22^	21	2018–2022	4169	2285	1884	67.2	EnSite™, CARTO^®^ 3	ATA and AF, AT/AFL recurrence post-blanking, and major complications	FPI and acute PVR, procedural time, PVI ablation number, RF time, and fluoroscopy time
Ravi et al. (2021)^23^	15	Until May 15, 2020	3718	2357	1361	65.5	EnSite™, CARTO^®^ 3	Freedom from atrial arrhythmia, acute PVR, total complications	Procedure duration, fluoroscopy duration, RFA duration
Waranugraha et al. (2021)^24^	13	Until Feb 2021	2901	1644	1257	55–84	EnSite™, CARTO^®^ 3	Recurrent AF, PVR, FPI	Procedure time, fluoroscopy time, ablation time, ETI
Li et al. (2021)^25^	7	N/A	2023	1244	779	64.8	EnSite™, CARTO^®^ 3	Recurrence of atrial arrhythmias, FPI	Procedural time, ablation time, fluoroscopy time, ETI
Li et al. (2022)^26^	22	N/A	3867	2393	1474	68.3	EnSite™, CARTO^®^ 3	FPI, acute PVR, freedom from AF, and freedom from atrial arrhythmia	Esophagus injury rate and major complication rate; effectiveness endpoints include complete PVI rate, total procedure time, PVI time, and PVI RFA time
Jin et al. (2022)^27^	17	Until Dec 2021	4934	2397	2537	69	EnSite™, CARTO^®^ 3	Total procedure duration, total RF duration, total fluoroscopy duration, first-pass isolation, freedom from atrial arrhythmia at 1 year, acute PVR, total complications	N/A

*Abbreviations:* AT/AFL, atrial tachycardia/atrial flutter; ATA, atrial tachyarrhythmia; CA, catheter ablation; ETI, esophageal thermal injury; FPI, first-pass pulmonary vein isolation; HPSD, high-power, short-duration; LPLD, low-power, long-duration; N/A, not available; PV, pulmonary vein; PVI, pulmonary vein isolation; PVR, pulmonary vein reconnection.

**Table 2:** Secondary Outcomes

Outcome	Effect Measure (RR or MD)	95% CI	*P* Value
FPI	RR, 1.18	1.04–1.35	.01
Total procedure time	MD, −22.64	−30.98 to −14.29	<.00001
Ablation number	MD, −4.89	−10.45 to 0.67	.09
Ablation time	MD, −11.76	−18.30 to −5.22	.0004
Fluoroscopy time	MD, −2.91	−3.99 to −1.83	<.00001
Acute PVR	RR, 0.51	0.45–0.58	<.00001

*Abbreviations:* CI, confidence interval; FPI, first-pass pulmonary vein isolation; MD, mean difference; PVI, pulmonary vein isolation; PVR, pulmonary vein reconnection; RR, risk ratio.

**Table 3:** Egger’s Test

Outcome	*Z* Value
ATA recurrence	0.0558
ETI	0.7511
Other major complications	0.9412

*Abbreviations:* ATA, atrial tachyarrhythmia; ETI, esophageal thermal injury.

**Supplementary Table S1:** Search Strategy

Database	Search Strategy	Number of Articles Found
PubMed	((“high-power”[All Fields] AND (“short”[All Fields] OR “shorts”[All Fields]) AND (“duration”[All Fields] OR “durations”[All Fields])) OR (“high”[All Fields] AND (“power, psychological”[MeSH Terms] OR (“power”[All Fields] AND “psychological”[All Fields]) OR “psychological power”[All Fields] OR “power”[All Fields] OR “powered”[All Fields] OR “powers”[All Fields] OR “powering”[All Fields]) AND (“short”[All Fields] OR “shorts”[All Fields]) AND (“duration”[All Fields] OR “durations”[All Fields])) OR “HPSD”[All Fields]) AND ((“low”[All Fields] AND (“power, Psychological”[MeSH Terms] OR (“power”[All Fields] AND “psychological”[All Fields]) OR “psychological power”[All Fields] OR “power”[All Fields] OR “powered”[All Fields] OR “powers”[All Fields] OR “powering”[All Fields]) AND “long”[All Fields] AND (“duration”[All Fields] OR “durations”[All Fields])) OR “LPLD”[All Fields]) AND (“radiofrequency ablation”[MeSH Terms] OR (“radiofrequency”[All Fields] AND “ablation”[All Fields]) OR “radiofrequency ablation”[All Fields] OR (“catheter ablation”[MeSH Terms] OR (“catheter”[All Fields] AND “ablation”[All Fields]) OR “catheter ablation”[All Fields]) OR (“ablate”[All Fields] OR “ablated”[All Fields] OR “ablates”[All Fields] OR “ablating”[All Fields] OR “ablation”[All Fields] OR “ablational”[All Fields] OR “ablations”[All Fields])) AND (“atrial fibrillation”[MeSH Terms] OR (“atrial”[All Fields] AND “fibrillation”[All Fields]) OR “atrial Fibrillation”[All Fields] OR (“atrial fibrillation”[MeSH Terms] OR (“atrial”[All Fields] AND “fibrillation”[All Fields]) OR “atrial fibrillation”[All Fields] OR “afib”[All Fields]))	17
Cochrane library	(((((high-power short duration) OR (high power short duration)) OR (HPSD)) AND ((low power long duration) OR (LPLD))) AND (((radiofrequency ablation) OR (catheter ablation)) OR (ablation))) AND ((atrial fibrillation) OR (Afib))	13
Embase	(((((high-power short duration) OR (high power short duration)) OR (HPSD)) AND ((low power long duration) OR (LPLD))) AND (((radiofrequency ablation) OR (catheter ablation)) OR (ablation))) AND ((atrial fibrillation) OR (Afib))	5

*Abbreviation:* MeSH, Medical Subject Heading.

**Supplementary Table S2:** Assessing the Methodological Quality of Systematic Reviews—AMSTAR 2

References	AMSTAR2 Items^a^																Overall Rating^b^
	1	2	3	4	5	6	7	8	9	10	11	12	13	14	15	16	
Kewcharoen et al. (2021)^17^	No	Yes	Yes	PY	Yes	Yes	No	Yes	Yes	No	Yes	No	No	Yes	No	Yes	Moderate
Khanra et al. (2022)^18^	Yes	Yes	Yes	Yes	Yes	Yes	Yes	Yes	Yes	Yes	Yes	No	Yes	Yes	No	Yes	Moderate
Chen et al. (2020)^19^	Yes	Yes	No	Yes	Yes	Yes	Yes	Yes	Yes	No	Yes	No	Yes	Yes	Yes	No	Moderate
Xu et al. (2022)^20^	Yes	Yes	No	Yes	Yes	Yes	Yes	Yes	Yes	No	Yes	No	Yes	Yes	Yes	No	Moderate
Parlavecchio et al. (2023)^21^	No	Yes	Yes	PY	Yes	Yes	No	Yes	Yes	No	Yes	No	No	Yes	No	Yes	Moderate
Kumar et al. (2023)^22^	Yes	Yes	No	Yes	Yes	Yes	Yes	Yes	Yes	No	Yes	No	Yes	Yes	Yes	No	Moderate
Ravi et al. (2021)^23^	Yes	Yes	Yes	Yes	Yes	Yes	Yes	Yes	Yes	Yes	Yes	No	Yes	Yes	No	Yes	Moderate
Waranugraha et al. (2021)^24^	Yes	Yes	No	Yes	Yes	Yes	Yes	Yes	Yes	No	Yes	No	Yes	Yes	Yes	No	Moderate
Li et al. (2021)^25^	No	Yes	Yes	PY	Yes	Yes	No	Yes	Yes	No	Yes	No	No	Yes	No	Yes	Moderate
Li et al. (2022)^26^	Yes	Yes	No	Yes	Yes	Yes	Yes	Yes	Yes	No	Yes	No	Yes	Yes	Yes	No	Moderate
Jin et al. (2022)^27^	Yes	Yes	No	Yes	Yes	Yes	Yes	Yes	Yes	Yes	Yes	Yes	Yes	No	Yes	No	Moderate
Total number of “yes” responses	8	11	5	8	11	11	8	11	11	3	11	1	8	10	6	5	

*Abbreviations:* PECO, Population, Exposure, Comparison, Outcome; PICO, Population, Intervention, Comparison, Outcome; PY, partial yes; RoB, risk of bias.

^a^AMSTAR items:
Did the research questions and inclusion criteria for the review include the components of PICO/PECO? Did the report of the review contain an explicit statement that the review methods were established prior to the conduct of the review and did the report justify any significant deviations from the protocol? Did the review authors explain their selection of the study designs for inclusion in the review? Did the review authors use a comprehensive literature search strategy? Did the review authors perform study selection in duplicate? Did the review authors perform data extraction in duplicate? Did the review authors provide a list of excluded studies and justify the exclusions? Did the review authors describe the included studies in adequate detail? Did the review authors use a satisfactory technique for assessing the RoB in individual studies that were included in the review? Did the review authors report on the sources of funding for the studies included in the review? If meta-analysis was performed, did the review authors use appropriate methods for statistical combination of results? If meta-analysis was performed, did the review authors assess the potential impact of RoB in individual studies on the results of the meta-analysis or other evidence synthesis? Did the review authors account for RoB in individual studies when interpreting/discussing the results of the review? Did the review authors provide a satisfactory explanation for, and discussion of, any heterogeneity observed in the results of the review? If they performed quantitative synthesis, did the review authors carry out an adequate investigation of publication bias (small-study bias) and discuss its likely impact on the results of the review? Did the review authors report any potential sources of conflict of interest, including any funding they received for conducting the review?

^b^Rating overall confidence in the results of the review:
High: No or one non-critical weakness: the systematic review provides an accurate and comprehensive summary of the results of the available studies that address the question of interest. Moderate: More than one non-critical weakness^c^: the systematic review has more than one weakness but no critical flaws. It may provide an accurate summary of the results of the available studies that were included in the review. Low: One critical flaw with or without non-critical weaknesses: the review has a critical flaw and may not provide an accurate and comprehensive summary of the available studies that address the question of interest. Critically low: More than one critical flaw with or without non-critical weaknesses: the review has more than one critical flaw and should not be relied on to provide an accurate and comprehensive summary of the available studies.

^c^Multiple non-critical weaknesses may diminish confidence in the review, and it may be appropriate to move the overall appraisal down from moderate to low confidence. Adapted from Shea et al. (2017).^10^

**Supplementary Table S3A:** Grade Assessment of the Meta-analyses and Systematic Reviews Included in Kewcharoen et al. (2021)^17^ to Compare High-power, Short-duration and Low-power, Long-duration Ablation for Atrial Tachycarrhythmias

Certainty Assessment							No. of Patients		Effect		Certainty	Importance
No. of Studies	Study Design	Risk of Bias	Inconsistency	Indirectness	Imprecision	Other Considerations	Intervention	Comparison	Relative (95% CI)	Absolute (95% CI)		
Recurrence of ATA												
10	Randomized and non-randomized studies	Serious	Serious	Not serious	Not serious	None	1393	881	**RR, 1.54** (0.99–2.40)	**0 fewer per 1000** (from 0 fewer to 0 fewer)	⨁⨁◯◯ Low	IMPORTANT
ETI												
10	Randomized and non-randomized studies	Serious	serious	Not serious	Not serious	None	1393	881	**RR, 0.35** (0.15–1.06)	**0 fewer per 1000** (from 0 fewer to 0 fewer)	⨁⨁◯◯ Low	CRITICAL

*Abbreviations:* ATA, atrial tachyarrhythmia; CI, confidence interval; ETI, esophageal thermal injury; HPSD, high-power, short-duration; LPLD, low-power, long-duration; RR, risk ratio.

**Supplementary Table S3B:** Grade Assessment of the Meta-analyses and Systematic Reviews Included in Khanra et al. (2022)^18^ to Compare High-power, Short-duration and Low-power, Long-duration Ablation for Atrial Tachycarrhythmias

Certainty Assessment							No. of Patients		Effect		Certainty	Importance
No. of Studies	Study Design	Risk of Bias	Inconsistency	Indirectness	Imprecision	Other Considerations	HPSD	LPLD	Relative (95% CI)	Absolute (95% CI)		
Recurrence of ATA												
20	Randomized and non-randomized studies	Serious	Serious	Not serious	Not serious	None	2136	1753	**RR, 0.66** (0.49–0.88)	**0 fewer per 1000** (from 0 fewer to 0 fewer)	⨁⨁◯◯ Low	IMPORTANT
ETI												
20	Randomized and non-randomized studies	Serious	Serious	Not serious	Not serious	None	2136	1753	**RR, 0.81** (0.32–2.10)	**0 fewer per 1000** (from 0 fewer to 0 fewer)	⨁⨁◯◯ Low	IMPORTANT

*Abbreviations:* ATA, atrial tachyarrhythmia; CI, confidence interval; ETI, esophageal thermal injury; HPSD, high-power, short-duration; LPLD, low-power, long-duration; RR, risk ratio.

**Supplementary Table S3C:** Grade Assessment of the Meta-analyses and Systematic Reviews Included in Chen et al. (2020)^19^ to Compare High-power, Short-duration and Low-power, Long-duration Ablation for Atrial Tachycarrhythmias

Certainty Assessment							No. of Patients		Effect		Certainty	Importance
No. of Studies	Study Design	Risk of Bias	Inconsistency	Indirectness	Imprecision	Other Considerations	HPSD	LPLD	Relative (95% CI)	Absolute (95% CI)		
Recurrence of ATA												
10	Randomized trials	Not serious	Not serious	Not serious	Not serious	None	1466	1001	**RR, 0.73** (0.58–0.91)	**0 fewer per 1000** (from 0 fewer to 0 fewer)	⨁⨁⨁⨁ High	CRITICAL
Other major complications												
10	Randomized trials	Not serious	Not serious	Not serious	Not serious	None	1466	1001	**RR, 0.75** (0.44–1.30)	**0 fewer per 1000** (from 0 fewer to 0 fewer)	⨁⨁⨁⨁ High	CRITICAL
ETI												
10	Randomized trials	Not serious	Not serious	Not serious	Not serious	None	1466	1001	**RR, 0.64** (0.17–2.39)	**0 fewer per 1000** (from 0 fewer to 0 fewer)	⨁⨁⨁⨁ High	CRITICAL

*Abbreviations:* ATA, atrial tachyarrhythmia; CI, confidence interval; ETI, esophageal thermal injury; HPSD, high-power, short-duration; LPLD, low-power, long-duration; RR, risk ratio.

**Supplementary Table S3D:** Grade Assessment of the Meta-analyses and Systematic Reviews Included in Xu et al. (2022)^20^ to Compare High-power, Short-duration and Low-power, Long-duration Ablation for Atrial Tachycarrhythmias

Certainty Assessment							No. of Patients		Effect		Certainty	Importance
No. of Studies	Study Design	Risk of Bias	Inconsistency	Indirectness	Imprecision	Other Considerations	HPSD	LPLD	Relative (95% CI)	Absolute (95% CI)		
Recurrence of ATA												
15	Randomized and non-randomized studies	Serious	Not serious	Not serious	Not serious	None	1856	1399	**RR, 0.54** (0.41–0.79)	**0 fewer per 1000** (from 0 fewer to 0 fewer)	⨁⨁⨁◯ Moderate	CRITICAL
Major complications												
15	Randomized and non-randomized studies	Serious	Not serious	Not serious	Not serious	None	1856	1399	**RR, 0.76** (0.27–2.16)	**0 fewer per 1000** (from 0 fewer to 0 fewer)	⨁⨁⨁◯ Moderate	CRITICAL
ETI												
15	Randomized and non-randomized studies	Serious	Not serious	Not serious	Not serious	None	1856	1399	**RR, 0.48** (0.30–0.77)	**0 fewer per 1000** (from 0 fewer to 0 fewer)	⨁⨁⨁◯ Moderate	CRITICAL

*Abbreviations:* ATA, atrial tachyarrhythmia; CI, confidence interval; ETI, esophageal thermal injury; HPSD, high-power, short-duration; LPLD, low-power, long-duration; RR, risk ratio.

**Supplementary Table S3E:** Grade Assessment of the Meta-analyses and Systematic Reviews Included in Parlavecchio et al. (2023)^21^ to Compare High-power, Short-duration and Low-power, Long-duration Ablation for Atrial Tachycarrhythmias

Certainty Assessment							No. of Patients		Effect		Certainty	Importance
No. of Studies	Study Design	Risk of Bias	Inconsistency	Indirectness	Imprecision	Other Considerations	HPSD	LPLD	Relative (95% CI)	Absolute (95% CI)		
Major complications												
5	Randomized trials	Not serious	Not serious	Not serious	Not serious	None	209	215	**RR, 1.45** (0.53–3.99)	**0 fewer per 1000** (from 0 fewer to 0 fewer)	⨁⨁⨁⨁ High	CRITICAL
ETI												
5	Randomized trials	Not serious	Not serious	Not serious	Not serious	None	209	215	**RR, 1.23** (0.38–3.98)	**0 fewer per 1000** (from 0 fewer to 0 fewer)	⨁⨁⨁⨁ High	CRITICAL

*Abbreviations:* ATA, atrial tachyarrhythmia; CI, confidence interval; ETI, esophageal thermal injury; HPSD, high-power, short-duration; LPLD, low-power, long-duration; RR, risk ratio.

**Supplementary Table S3F:** Grade Assessment of the Meta-analyses and Systematic Reviews Included in Kumar et al. (2023)^22^ to Compare High-power, Short-duration and Low-power, Long-duration Ablation for Atrial Tachycarrhythmias

Certainty Assessment							No. of Patients		Effect		Certainty	Importance
No. of Studies	Study Design	Risk of Bias	Inconsistency	Indirectness	Imprecision	Other Considerations	HPSD	LPLD	Relative (95% CI)	Absolute (95% CI)		
Recurrence of ATA												
21	Randomized and non-randomized studies	Serious	Not serious	Not serious	Not serious	None	2285	1884	**RR, 0.62** (0.50–0.78)	**0 fewer per 1000** (from 0 fewer to 0 fewer)	⨁⨁⨁◯ Moderate	IMPORTANT
Major complications												
21	Randomized and non-randomized studies	Serious	Not serious	Not serious	Not serious	None	2285	1884	**RR, 0.66** (0.29–1.50)	**0 fewer per 1000** (from 0 fewer to 0 fewer)	⨁⨁⨁◯ Moderate	IMPORTANT
ETI												
21	Randomized and non-randomized studies	Serious	Not serious	Not serious	Not serious	None	2285	1884	**RR, 0.78** (0.58–1.04)	**0 fewer per 1000** (from 0 fewer to 0 fewer)	⨁⨁⨁◯ Moderate	IMPORTANT

*Abbreviations:* ATA, atrial tachyarrhythmia; CI, confidence interval; ETI, esophageal thermal injury; HPSD, high-power, short-duration; LPLD, low-power, long-duration; RR, risk ratio.

**Supplementary Table S3G:** Grade Assessment of the Meta-analyses and Systematic Reviews Included in Ravi et al. (2021)^23^ to Compare High-power, Short-duration and Low-power, Long-duration Ablation for Atrial Tachycarrhythmias

Certainty Assessment							No. of Patients		Effect		Certainty	Importance
No. of Studies	Study Design	Risk of Bias	Inconsistency	Indirectness	Imprecision	Other Considerations	HPSD	LPLD	Relative (95% CI)	Absolute (95% CI)		
Recurrence of ATA												
15	Randomized and non-randomized studies	Serious	Serious	Not serious	Not serious	None	2357	1361	**RR, 1.44** (1.10 to 1.90)	**0 fewer per 1000** (from 0 fewer to 0 fewer)	⨁⨁◯◯ Low	IMPORTANT
Major complications												
15	Randomized and non-randomized studies	Serious	Serious	Not serious	Not serious	None	2357	1361	**RR, 0.83** (0.63 to 1.09)	**0 fewer per 1000** (from 0 fewer to 0 fewer)	⨁⨁◯◯ Low	IMPORTANT

*Abbreviations:* ATA, atrial tachyarrhythmia; CI, confidence interval; HPSD, high-power, short-duration; LPLD, low-power, long-duration; RR, risk ratio.

**Supplementary Table S3H:** Grade Assessment of the Meta-analyses and Systematic Reviews Included in Waranugraha et al. (2021)^24^ to Compare High-power, Short-duration and Low-power, Long-duration Ablation for Atrial Tachycarrhythmias

Certainty Assessment							No. of Patients		Effect		Certainty	Importance
No. of Studies	Study Design	Risk of Bias	Inconsistency	Indirectness	Imprecision	Other Considerations	HPSD	LPLD	Relative (95% CI)	Absolute (95% CI)		
Recurrence of ATA												
13	Randomized and non-randomized studies	Serious	Not serious	Not serious	Not serious	None	1644	1257	**RR, 0.72** (0.54–0.96)	**0 fewer per 1000** (from 0 fewer to 0 fewer)	⨁⨁⨁◯ Moderate	IMPORTANT
ETI												
13	Randomized and non-randomized studies	Serious	Not serious	Not serious	Not serious	None	1644	1257	**RR, 0.75** (0.59–0.94)	**0 fewer per 1000** (from 0 fewer to 0 fewer)	⨁⨁⨁◯ Moderate	IMPORTANT

*Abbreviations:* ATA, atrial tachyarrhythmia; CI, confidence interval; ETI, esophageal thermal injury; HPSD, high-power, short-duration; LPLD, low-power, long-duration; RR, risk ratio.

**Supplementary Table S3I::** Grade Assessment of the Meta-analyses and Systematic Reviews Included in Li et al. (2021)^25^ to Compare High-power, Short-duration and Low-power, Long-duration Ablation for Atrial Tachycarrhythmias

Certainty Assessment							No. of Patients		Effect		Certainty	Importance
No. of Studies	Study Design	Risk of Bias	Inconsistency	Indirectness	Imprecision	Other Considerations	HPSD	LPLD	Relative (95% CI)	Absolute (95% CI)		
Recurrence of ATA												
7	Randomized and non-randomized studies	Serious	Serious	Not serious	Not serious	None	1244	779	**RR, 0.70** (0.50–0.98)	**0 fewer per 1000** (from 0 fewer to 0 fewer)	⨁⨁◯◯ Low	CRITICAL
ETI												
7	Randomized and non-randomized studies	Serious	Serious	Not serious	Not serious	None	1244	779	**RR, 0.75** (0.44–1.30)	**0 fewer per 1000** (from 0 fewer to 0 fewer)	⨁⨁◯◯ Low	CRITICAL

*Abbreviations:* ATA, atrial tachyarrhythmia; CI, confidence interval; ETI, esophageal thermal injury; HPSD, high-power, short-duration; LPLD, low-power, long-duration; RR, risk ratio.

**Supplementary Table S3J:** Grade Assessment of the Meta-analyses and Systematic Reviews Included in Li et al. (2022)^26^ to Compare High-power, Short-duration and Low-power, Long-duration Ablation for Atrial Tachycarrhythmias

Certainty Assessment							No. of Patients		Effect		Certainty	Importance
No. of Studies	Study Design	Risk of Bias	Inconsistency	Indirectness	Imprecision	Other Considerations	HPSD	LPLD	Relative (95% CI)	Absolute (95% CI)		
Recurrence of ATA												
22	Randomized and non-randomized studies	Serious	Not serious	Not serious	Not serious	None	2393	1474	**RR, 1.17** (1.07–1.27)	**0 fewer per 1000** (from 0 fewer to 0 fewer)	⨁⨁⨁◯ Moderate	CRITICAL
Other major complications												
22	Randomized and non-randomized studies	Serious	Not serious	Not serious	Not serious	None	2393	1474	**RR, 0.82** (0.30–2.26)	**0 fewer per 1000** (from 0 fewer to 0 fewer)	⨁⨁⨁◯ Moderate	CRITICAL
ETI												
22	Randomized and non-randomized studies	Serious	Not serious	Not serious	Not serious	None	2393	1474	**RR, 0.99** (0.31–3.13)	**0 fewer per 1000** (from 0 fewer to 0 fewer)	⨁⨁⨁◯ Moderate	CRITICAL

*Abbreviations:* ATA, atrial tachyarrhythmia; CI, confidence interval; ETI, esophageal thermal injury; HPSD, high-power, short-duration; LPLD, low-power, long-duration; RR, risk ratio.

**Supplementary Table S3K:** Grade Assessment of the Meta-analyses and Systematic Reviews Included in Jin et al. (2022)^27^ to Compare High-power, Short-duration and Low-power, Long-duration Ablation for Atrial Tachycarrhythmias

Certainty Assessment							No. of Patients		Effect		Certainty	Importance
No. of Studies	Study Design	Risk of Bias	Inconsistency	Indirectness	Imprecision	Other Considerations	HPSD	LPLD	Relative (95% CI)	Absolute (95% CI)		
Recurrence of ATA												
17	Randomized and non-randomized studies	Serious	Not serious	Not serious	Not serious	None	2397	2537	**RR, 1.48** (1.12–1.94)	**0 fewer per 1000** (from 0 fewer to 0 fewer)	⨁⨁⨁◯ Moderate	CRITICAL
Other major complications												
17	Randomized and non-randomized studies	Serious	Not serious	Not serious	Not serious	None	2397	2537	**RR, 0.95** (0.72–1.25)	**0 fewer per 1000** (from 0 fewer to 0 fewer)	⨁⨁⨁◯ Moderate	CRITICAL

*Abbreviations:* ATA, atrial tachyarrhythmia; CI, confidence interval; ETI, esophageal thermal injury; HPSD, high-power, short-duration; LPLD, low-power, long-duration; RR, risk ratio.

**Supplementary Table S4:** Newcastle–Ottawa Scale

Study	Selection				Comparability	Outcomes			Total
	Representativeness of the Exposed Cohort	Selection of the Non-exposed Cohort	Ascertainment of Exposure	Demonstration That Outcome of Interest Was Not Present at Start of Study	Comparability of Cohorts on the Basis of the Design or Analysis	Assessment of Outcome	Was Follow-up Long Enough for Outcomes to Occur	Adequacy of Follow-up of Cohorts	
Vassallo (2020)	*	*	*	*	**	*	*	*	*********
Yavin et al. (2020)	*	*	*	*	*	*	*	*	********
Yazaki (2020)	*	*	*	*	*	*	*	*	********
Kottmaier (2020)	*	*	*	-	*	*	*	*	******
Kaneshiro (2020)	*	*	*	*	**	*	*	*	*********
Ejima (2020)	*	*	*	*	*	*	*	*	********
Castrejón (2020)	*	*	*	*	*	*	*	*	********
Bunch (2020)	*	*	*	-	*	*	*	*	******
Vassallo et al. (2019)	*	*	*	*	**	*	*	*	*********
Pambrun (2019)	*	*	*	*	*	*	*	*	********
Okamatsu (2019)	*	*	*	-	*	*	*	*	******
Berte (2019)	*	*	*	*	**	*	*	*	*********
Baher (2018)	*	*	*	*	*	*	*	*	********
Hansom (2021)	*	*	*	-	*	*	*	*	******
Sousa (2023)	*	*	*	*	**	*	*	*	*********
Sallo (2022)	*	*	*	*	*	*	*	*	********
Cheng (2022)	*	*	*	-	*	*	*	*	******
Vassallo (2022)	*	*	*	*	**	*	*	*	*********
Nilsson (2006)	*	*	*	*	*	*	*	*	********
Yamada (2006)	*	*	*	*	**	*	*	*	*********
Dhillon (2018)	*	*	*	*	*	*	*	*	********
Kyriakopoulou (2018)	*	*	*	*	*	*	*	*	********
Leo (2020)	*	*	*	-	*	*	*	*	******
Dikdan (2021)	*	*	*	*	**	*	*	*	*********
Tilz (2021)	*	*	*	*	*	*	*	*	********
Lee (2021)	*	*	*	*	**	*	*	*	*********

This table thoroughly assesses the quality of observational studies included in the meta-analyses that were pooled for this meta-analysis using the Newcastle–Ottawa scale. The evaluation delineates a spectrum of study quality, ranging from fair to good. In the Newcastle–Ottawa scale, an asterisk (*) signifies a point awarded for meeting specific criteria, with good quality defined as 3 or 4 stars in the selection domain AND 1 or 2 star(s) in the comparability domain AND 2 or 3 stars in the outcome/exposure domain. Fair quality is characterized by 2 stars in the selection domain, 1 OR 2 star(s) in the comparability domain, AND 2 or 3 stars in the outcome/exposure domain. Poor quality is indicated by 0 or 1 star(s) in the selection domain, 0 stars in the comparability domain, OR 0 or 1 star(s) in the outcome/exposure domain.
